# Plasma cell differentiation is controlled by multiple cell division-coupled epigenetic programs

**DOI:** 10.1038/s41467-018-04125-8

**Published:** 2018-04-27

**Authors:** Christopher D. Scharer, Benjamin G. Barwick, Muyao Guo, Alexander P. R. Bally, Jeremy M. Boss

**Affiliations:** 1 Department of Microbiology and Immunology, Emory University, Atlanta, GA 30322 USA; 2 Present Address: Department of Hematology and Medical Oncology, Emory University, Atlanta, GA 30322 USA; 3 Present Address: Xiangya School of Medicine, Central South University, Changsha, Hunan 410008 China

## Abstract

The genomic loci associated with B cell differentiation that are subject to transcriptional and epigenetic regulation in vivo are not well defined, leaving a gap in our understanding of the development of humoral immune responses. Here, using an in vivo T cell independent B cell differentiation model, we define a cellular division-dependent *cis*-regulatory element road map using ATAC-seq. Chromatin accessibility changes correlate with gene expression and reveal the reprogramming of transcriptional networks and the genes they regulate at specific cell divisions. A subset of genes in naive B cells display accessible promoters in the absence of transcription and are marked by H3K27me3, an EZH2 catalyzed repressive modification. Such genes encode regulators of cell division and metabolism and include the essential plasma cell transcription factor Blimp-1. Chemical inhibition of EZH2 results in enhanced plasma cell formation, increased expression of the above gene set, and premature expression of Blimp-1 ex vivo. These data provide insights into cell-division coupled epigenetic and transcriptional processes that program plasma cells.

## Abstract (web summary)

During B cell differentiation, the role of different genomic loci in transcriptional and epigenetic regulation in vivo is not well defined. Here the authors use an in vivo B cell differentiation model to map cellular division-dependent *cis*-regulatory element road map with ATAC-seq.

## Introduction

The differentiation of B cells to plasma cells is an essential process for humoral immunity. Following ligand engagement with Toll-like receptors or the B cell receptor, B cells differentiate into either rapidly proliferating plasmablasts and post-mitotic plasma cells or memory B cells. Differentiation requires changes in metabolism to facilitate antibody secretion^1^ and expansion in RNA content and transcription capacity^2, 3^. The DNA-binding factors that program the B cell and plasma cell fates have been extensively studied and portray two mutually exclusive transcription factor networks, which include the factors BCL6, BACH2, Blimp-1 and others, that function to repress the alternate fate^4^. While the genetic associations between these factors have been revealed, data is lacking that defines the entire *cis*-regulatory element network that regulates B cell to plasma cell differentiation.

Recently, it is becoming clear that the epigenome is remodeled in tandem with changes in gene expression programs during B cell differentiation. The unique transcriptomes of B cells and plasma cells have been defined for multiple stimuli and species^2, 5, 6^. Epigenetically, plasma cells possess distinct DNA methylation landscapes from their naive counterparts^6, 7^, and in vivo models that integrate cellular division with epigenetic and transcriptional changes demonstrate a linkage between changes in DNA methylation and cellular division^2^. These data revealed that few CpG loci gained DNA methylation, suggesting that other epigenetic processes facilitate gene repression during plasma cell formation^2^. One such mechanism may lie with the polycomb repressor complex, which serves to epigenetically silence gene expression through modification of chromatin^8, 9^. EZH2, a catalytic subunit of the polycomb complex, deposits the repressive histone modification H3K27me3 and is essential during B cell development^10^ and during the germinal center reaction^11–13^. BCL6^14^ and Blimp-1^15^ differentially recruit EZH2 to distinct regions of DNA to repress genes that dictate plasma cell and B cell fates, respectively, and demonstrate that sequence specific DNA-binding proteins can recruit epigenetic enzymes to distinct loci. Moreover, such studies provide a potential mechanism for how changes in transcription factor networks facilitate remodeling of the epigenome during differentiation.

Because, epigenetic mechanisms can function to restrict or promote the accessibility of DNA to sequence-specific binding proteins, identifying differentially accessible regions between two cell types can reveal regulatory elements that function to regulate gene expression and thus cell fate decisions. The assay for transposase accessible chromatin-sequencing (ATAC-seq) is a sensitive tool that can detect accessible regions in the genome, including promoters and distal regulatory elements^16, 17^. The analysis of transcription factor DNA sequence motifs in regions that display altered accessibility provides an indication of which transcription factor families and pathways may be involved in the regulation of gene expression and differentiation. Here, ATAC-seq was applied to an in vivo model of B cell differentiation^2^ to define the *cis*-regulatory changes that occur at distinct differentiation stages defined by cellular divisions. We identify a unique set of promoters that exhibit increased accessibility in the absence of gene expression in undivided B cells. A subset of these primed promoters are enriched for H3K27me3 and chemical inhibition of EZH2 enhances expression of primed genes and increases plasma cell formation ex vivo. Together, these data provide a role for EZH2 dependent control of B cell fate that is orchestrated and regulated in a division-dependent manner.

## Results

### B cell differentiation remodels the chromatin landscape

**Fig. 1:**
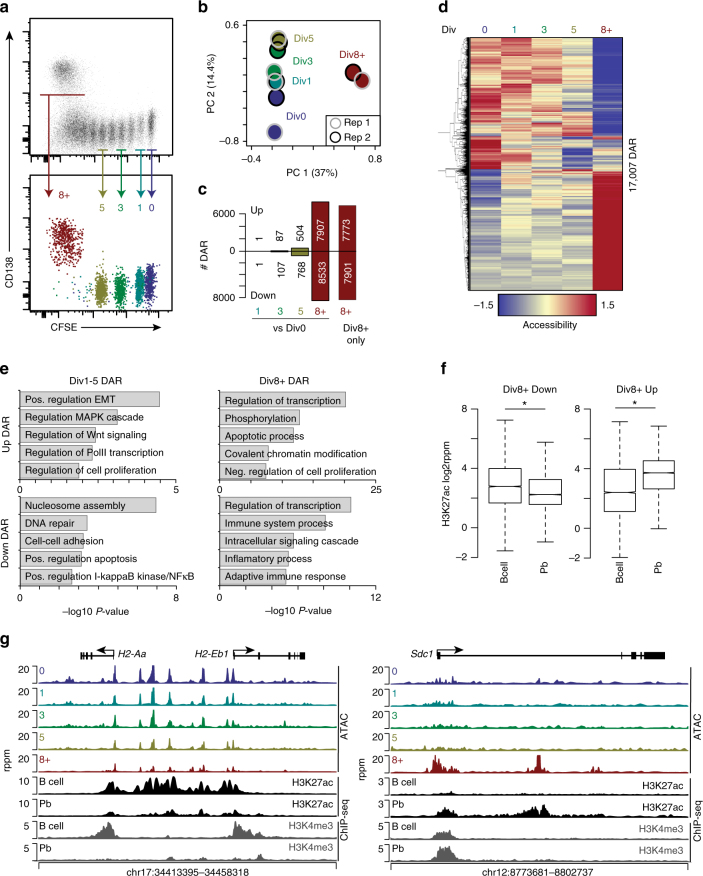
B cell differentiation is coupled to division-specific changes in chromatin accessibility. **a** Splenic CD45.1 B cells were labeled with CFSE and transferred to a µMT (CD45.2) mouse. Twenty-four hours later the mice were inoculated with LPS and splenic B cells were harvested three days post inoculation. Flow cytometry of CD45.1 cells (top) and CSFE sorted populations (bottom) used in this study. **b** PCA plot of 60,766 accessible loci across all samples. **c** Bar plot quantifying the number of DAR at each division compared to Div0 B cells that either gained (up) or lost (down) accessibility or those DAR that are specific for Div8+. **d** Heat map of ATAC-seq accessibility changes across each division for all significantly differentially accessible regions (DAR). **e** Gene ontology analysis of up and down DAR from Div1-5 and Div8+. **f** Quantification of H3K27ac levels at Div8+ up and down DAR in naive B cells (nB) and plasmablasts (Pb). Boxplot center line indicates data median, lower and upper bounds of boxes the 1st and 3rd quartile ranges, and whiskers the upper and lower ranges of the data. *P-value < 2.6e−16 determined by two-tailed Student’s *T*-test. **g** ATAC-seq and ChIP-seq profile for the MHCII and *Sdc1* gene loci is plotted. ATAC-seq and ChIP-seq data are average of two biological replicates at each division or cell type. PC principal component

An in vivo model system was developed that allowed the analysis of molecular and epigenetic events to be ascertained with respect to the number of cellular divisions that occur during B cell to plasma cell differentiation^2^. Acquisition of the surface molecule CD138 in tandem with decreased levels of B220 can be used as a marker for plasmablast and plasma cell formation^18^. Previous in vivo data indicated that while transcriptional gains and losses occurred, DNA methylation was predominantly lost in a division-dependent manner during type 1 T cell independent B cell differentiation. These data also suggested that other epigenetic mechanisms participate in silencing gene expression in the absence of de novo DNA methylation. To define *cis*-elements linked to cellular division-and gene regulation during B cell differentiation in vivo, B cells were CFSE labeled adoptively transferred into μMT mice^19^, and subsequently inoculated with LPS to induce B cell differentiation. Three days post-LPS challenge, five distinct divisions were isolated by FACS, representing undivided (Div0); activated, dividing B cells (Div1, 3, and 5); and cells that had differentiated and acquired the plasma cell surface marker CD138 (Div8+) (Fig. 1a). Thus, Div8+ cells are plasmablasts (Pb) or plasma cells but are referred to as Div8+ here to maintain a division-centric nomenclature. ATAC-seq was applied to the cells from each division, and high-resolution chromatin accessibility maps were generated. Principal component analysis (PCA) of the 60,766 accessible loci detected across all samples indicated the major source of variation was between cells that were CD138^−^ (Div0-5) and CD138^+^ (Div8+) (Fig. 1b). The second major variation, PC2, separated Div0 through 5 linearly by division, suggesting that progressive changes in accessibility occurred between each of the cellular divisions.

Differentially accessible regions (DAR) were determined for all samples compared to the undivided B cells (Div0). In total, 17,007 loci were differentially accessible with roughly equal numbers of loci that gained or lost accessibility (Fig. 1c, d, Supplementary Data 1). A relatively small number of DARs appeared at Div3 and Div5 and in agreement with the PCA, the greatest number of DARs occurred at Div8+. Furthermore, 95% (15,674/16,440) of the Div8+ DAR were specific for that division and correlated with the formation of plasma cells and the acquisition of CD138. To identify the functions of genes surrounding DAR, each DAR was annotated to the closest gene and gene ontology (GO) analysis was performed for early divisions representing the proliferative phase (Div1-5) and Div8+ representing differentiation. During the early divisions, gains in accessibility were associated with genes that function in endothelial mesenchymal transition (e.g., *Ezh2*, *Tgfbr2*), signaling cascades (e.g., *Pik3r1*, *Smad4*), and cellular proliferation (e.g., *Ccnd1*); while increased accessibility in Div8+ occurred in genes encoding transcriptional regulators (e.g., *Prdm1*, *Irf4*) and regulators of apoptosis (e.g., *Bcl2, Mcl1*). Decreases in accessibility were associated with down regulation of B cell lineage genes (Immune system process: e.g., *Irf8, H2-Aa*) and positive regulators of NF-κB signaling (e.g., *Traf3ip2*, *Rel*).

To validate that DAR marked regulatory elements at loci that underwent changes in epigenetic modifications ChIP-seq was performed for H3K27ac and H3K4me3 in naive splenic B cells and Pb formed three days following LPS inoculation as above. Consistent with a function in B cells, DAR that lost accessibility between Div8+ and Div0 demonstrated a significant loss in H3K27ac signal between B cells and Pb (Fig. 1f). These regions are highlighted by the MHCII genes *H2-Aa* and *H2-Eb1* which are silenced during B cell differentiation to Pb. Multiple DAR lost accessibility and demonstrated a concurrent loss in H3K27ac throughout the locus and H3K4me3 at the promoter regions (Fig. 1g). Conversely, Div8+ DAR that gained accessibility were significantly enriched for H3K27ac in Pb and are exemplified by the *Sdc1* locus.

### Association of DNA methylation and accessibility changes

**Fig. 2:**
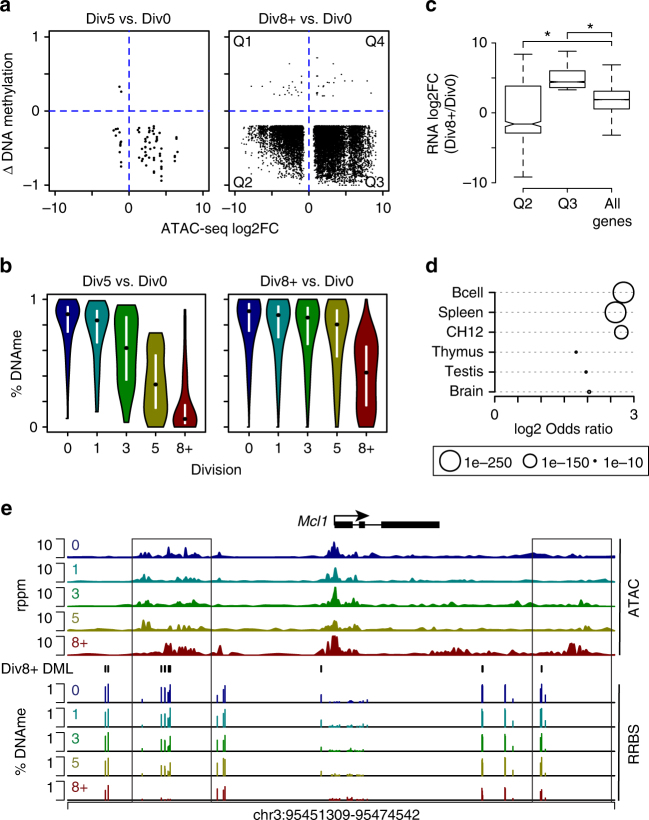
Chromatin accessibility changes are associated with losses in DNA methylation. **a** The change in DNA methylation versus the change in chromatin accessibility for regions that overlap at the indicated division was plotted. DNA methylation data was previously reported^2^. **b** Violin plot describing the percent DNA methylation at each division for CpGs that are plotted in **a**. The mean methylation is indicated by a black dot and white lines denote s.d. **c** Box plot displaying the fold change in gene expression between Div8+ versus Div0 for genes that map to quadrants Q2 and Q3 from **a** compared to the change at all genes. Boxplot center line indicates data median, lower and upper bounds of boxes the 1st and 3rd quartile ranges, and whiskers the upper and lower ranges of the data. *P-value < 2e−16 as determined by Wilcoxon Rank Sum test. **d** The odds ratio (log2 scale) for enhancers from the indicated tissue with loci that are denoted as DAR and DML in **a** between Div8+ versus Div0. Significance was determined by a Fisher’s exact test. **e** ATAC-seq and DNA methylation profile for the *Mcl1* locus is plotted. Boxed regions are B cell lineage enhancers

During LPS-induced B cell differentiation, approximately 10% of all CpG change their methylation status, with nearly all losing their methylation. Most hypomethylated CpGs occurred at later divisions and were located within B cell enhancers^2^. To determine the relationship between DNA methylation and chromatin accessibility, the overlap of DAR and differentially methylated loci (DML) at Div5 and Div8+ was computed and the correlation of the datasets was assessed. At Div5, an inverse relationship was observed such that sites that lost DNA methylation gained accessibility (Fig. 2a). At Div8+, as at Div5, nearly all the DAR that contained a DML lost methylation. DML that overlapped DAR at Div5 lost DNA methylation earlier than those that overlapped a Div8+ DAR (Fig. 2b), suggesting an ordered differentiation process through the divisions. Gene expression changes linked to DAR and DML were also explored. Demethylated loci in DAR that gained accessibility between Div0 and Div8+ (Fig. 2a, Q3) were associated with a set of genes that all significantly gained expression compared to all genes (Fig. 2c). Conversely, DML that lost methylation and mapped to DAR that also lost accessibility (Fig. 2a, Q2) were associated with genes that on average decreased expression (Fig. 2c), indicating that mechanisms involved in gene repression were manifested in changes in chromatin accessibility but not DNA methylation. For loci that contained both DAR and DML, the overlap with B cell lineage-specific enhancer landscapes previously determined^2^, as well as similar and distant tissues was computed. While the enhancers at distant tissues (Brain and Testis) exhibited an overlap, B cells, splenic enhancers, and the B cell lymphoma CH12 cell line had a higher odds ratio and were exponentially more statistically significant. For example, the *Mcl1* locus, which is upregulated at Div8+, contained two DAR that gained accessibility at Div8+ and contained demethylated DML that occurred at B cell lineage regulatory regions (Fig. 2d). These data showed that DML and DAR fall within B cell enhancers and that both increases and decreases in chromatin accessibility correlate with gene expression.

### DAR encode lineage-specific transcription factor footprints

**Fig. 3:**
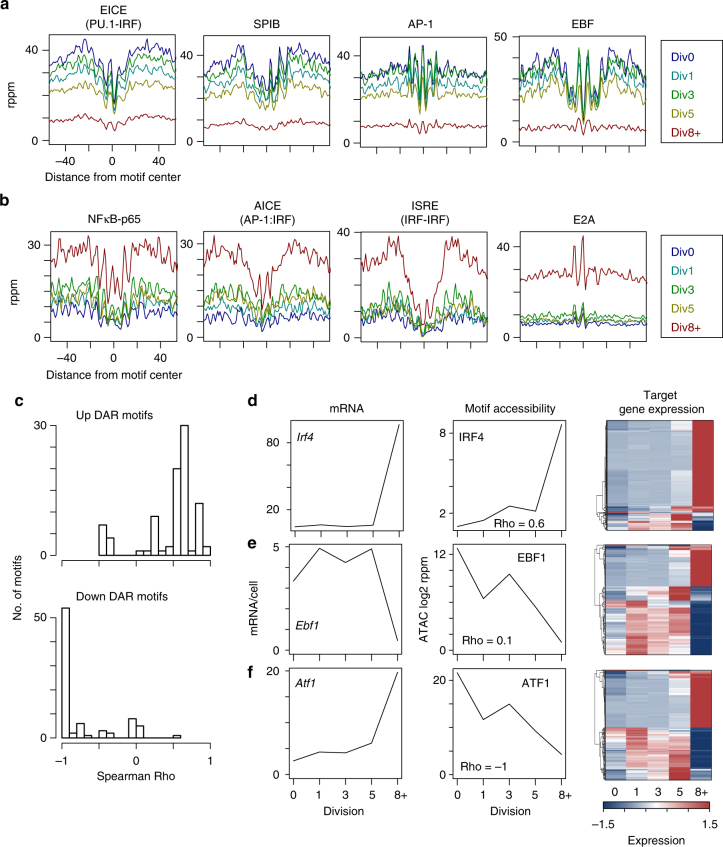
Transcription factor networks program accessibility changes. ATAC-seq derived transcription factor footprints at each division for the indicated motif that was enriched in either down (**a**) or up (**b**) DAR is shown. **c** For each transcription factor binding motif, the average accessibility was calculated and correlated with the expression of the transcription factor across all divisions. Correlations were plotted as Spearman Rho coefficient vs. number of motifs that lost (Down) or gained (Up) accessibility. **d**–**f** For select motifs from **c** the expression at each division (left), the accessibility of the motif (middle), and the expression of the predicted target genes (right) is plotted. The Spearman Rho coefficient for each comparison is indicated

Transcription factor DNA-binding motifs enriched in DAR were identified to determine the putative transcription factors/families affected by chromatin accessibility changes at each division. The ETS or the composite ETS:IRF family motifs were prevalent across all divisions in DAR that lost accessibility (Supplementary Fig. 1a), suggesting that at a chromatin level, accessibility at these sites is lost to extinguish the B cell program. In ATAC-seq datasets, a discrete footprint reflecting variations in accessibility across a transcription factor motif and the surrounding chromatin can be computed^16, 17^. Base-pair resolution footprints of the ETS family factor SPIB and composite ETS:IRF motif bound by PU.1:IRF8, which negatively regulate plasma cell formation^20^, were analyzed at each division. Both motifs progressively lost accessibility through each division, suggesting that their respective factors no longer functioned at these loci (Fig. 3a). In agreement with the rearrangement of the chromatin architecture upon B cell differentiation^21^, CTCF motifs were enriched at loci that both lost and gained accessibility (Supplementary Fig. 1). Sites that increased accessibility were enriched for factors that control plasma cell differentiation and function including IRF, AP-1, OCT2 motifs, and composite elements including the AICE (IRF:AP-1) and ISRE (IRF:IRF) motifs (Fig. 3b, Supplementary Fig. 1b)^4, 22–24^. Thus, DAR were enriched for transcription factor motifs that function in the differentiation of B cells to plasma cells.

Intriguingly, accessibility changes at the above transcription factor binding motifs occurred in a progressive manner through the distinct cell divisions that reflects activation and differentiation as follows. In the early divisions, changes at NF-κB, AP-1, and composite IRF:AP-1 motifs changed accessibility first; consistent with a role for these factors in activation and proliferation^24, 25^. Conversely, IRF4, IRSE, and the E-box factor sites E2A and TCF12 (HEB) become uniquely accessible at Div8+ (Fig. 3b, Supplementary Fig. 1b). Binding sites for the B cell development factor, EBF, uniquely lost accessibility at Div8+, suggesting that its role in maintaining a B cell fate was no longer required. As above, further analyses of these data revealed remarkable changes in accessibility footprints across the divisions with the greatest changes at Div8+ (Fig. 3a, b). IRF4 has been demonstrated to interact with distinct binding partners during B cell differentiation with the IRF:AP-1 heterodimer (AICE motif) active early and the IRF:IRF homodimer binding to ISRE motifs functioning at later time points^24^. These data support the notion that a hierarchy of transcription factors function to regulate the proliferation/activation versus the differentiation phase of B cell differentiation, and that this hierarchy is linked to cell division.

### Correlation of motif accessibility and expression dynamics

The expression of transcription factors themselves is dynamic during B cell differentiation and this could alter accessibility at cognate binding motifs and/or the expression of target genes. This relationship was explored by first defining a set of 99 transcription factors whose expression was detected in the division RNA-seq dataset^2^ (expressed at >1 mRNA/cell) and contained binding motifs in DAR (Supplementary Fig. 2, Supplementary Data 2). Such factors included *Spi1* (PU.1)*, Spib*, *Ebf1*, *Tbx21*, *Irf4*, and *Irf1*, with each having distinct patterns of expression across the divisions. For DAR that gained or lost accessibility, the correlation between the change in accessibility at each motif and the expression of its respective transcription factor was calculated across the divisions using the Spearman Rho coefficient. For the 85% (65/76) of motifs that gained accessibility there was a positive correlation with an increase in the transcription factor’s expression (Fig. 3c, top). For example, *Irf4* expression increased sharply at Div8+ and correlated with the largest increase in accessibility at IRF4 motifs (Fig. 3d). Such transcription factors and their target genes are likely to be activating. Examination of the expression of the IRF4 target gene set shows that a great majority of these target genes increase in expression in a manner that correlates with IRF4 gene expression (Fig. 3d). Similarly, there was a positive correlation with the loss of transcription factor expression for the 7% of DAR motifs that lost accessibility. This suggests that the loss of the transcription factor binding may have influenced accessibility. For example, sites containing EBF1 motifs lost accessibility in a step-wise manner in congruence with decreased *Ebf1* expression (Fig. 3e). Fifty-eight percent of EBF1 target genes showed decreases in expression (Fig. 3e).

Additionally, negative correlations (68/82 (83%)), such as that for ATF1, were observed in which the expression of a transcription factor increased over the divisions, yet accessibility at its cognate site was decreased (Fig. 3c, f). This suggests that the binding of the transcription factor may function differently at different sites. For example, chromatin surrounding ATF1 binding motifs lost accessibility while *Atf1* mRNA expression increased and ATF1 target genes were both induced and repressed (Fig. 3f). In response to TLR4 signaling the ATF family member ATF3 has been shown to repress *Il6* and *Il12b* transcription^26^. ATF1 may be performing a similar repressive function at genes that increase expression when ATF1 motif accessibility is lost. These data suggest a mechanism whereby decreases in chromatin accessibility could occur by active processes and not simply driven by the absence or presence of a factor, and that it is likely that accessibility at such sites is controlled by additional factors or different members of the same factor binding family.

### Distal element accessibility correlates with gene expression

**Fig. 4:**
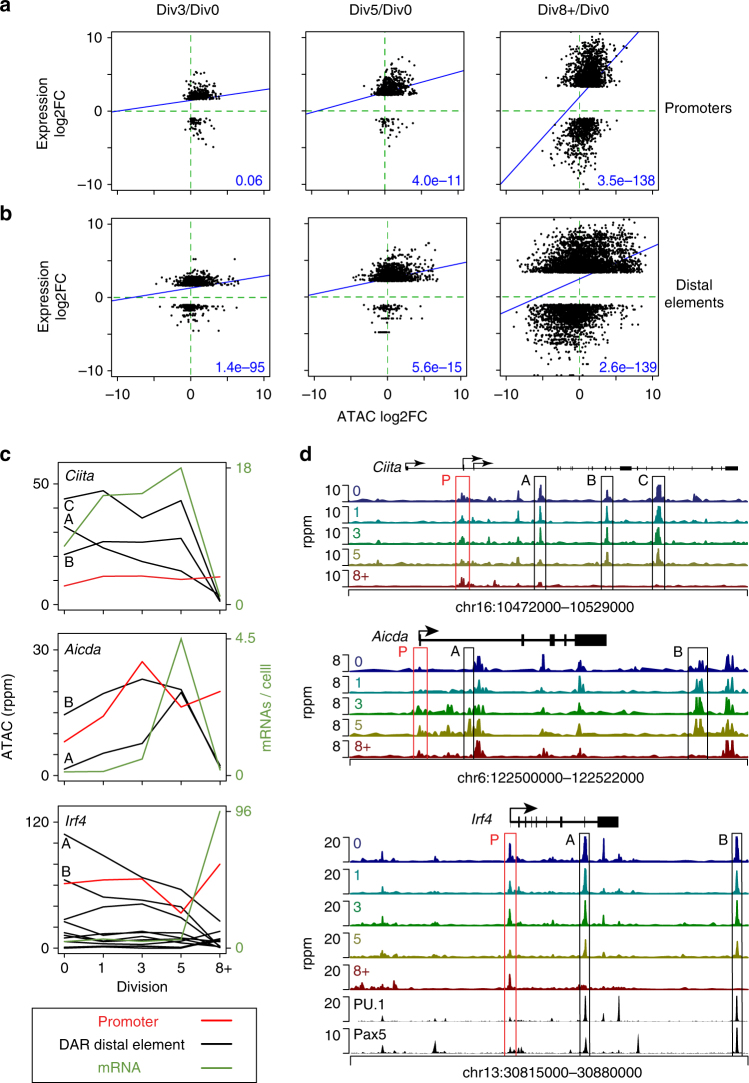
Coordinated changes in gene expression and distal *cis*-element accessibility. Gene expression and chromatin accessibility changes between the indicated division comparison for **a** promoters and **b** distal elements are shown as scatter plots. The significance of the linear regression is indicated. **c** For the indicated gene, the accessibility of all distal element DAR (black line) and the promoter (red line) are plotted with respect to each division. Overlaid on the data is the mRNA expression profile (green) for the same cellular divisions. **d** ATAC-seq accessibility profile for each gene plotted in **c**. For *Irf4*, previously reported B cell data for PU.1^35^ and Pax5^36^ are plotted. rppm, reads per peak per million

DNA methylation changes at B cell specific enhancers were associated with changes in gene expression during B cell differentiation^2^. In a similar manner, changes in histone modifications at enhancers correlated with gene expression more robustly than at promoters in other immune cell types^27–29^. This suggests that accessibility differences at enhancers may better reflect gene expression changes. To address this supposition, enhancer and promoter accessibility were separately correlated with gene expression. Here, promoters were defined as gene proximal regions located between −2500 to +500 bp surrounding a TSS. Distal elements were defined simply as accessible regions outside of the proximal promoter regions and within 100 kb of the nearest TSS. Promoter accessibility showed significant correlations with gene expression at Div5 and Div8+. At each division, promoters largely gained accessibility even if gene expression was up or down with a small subset of promoters demonstrating accessibility losses (Fig. 4a). *Ccr7* and *Egr1* are examples of genes where promoter accessibility changes correlated with expression changes (Supplementary Fig. 3a, b). In contrast to promoters, distal elements demonstrated a larger dynamic range in accessibility changes and a highly significant correlation with gene expression at all divisions (Fig. 4b). While there was no difference in correlation between promoters and distal elements at later divisions, Div3 showed a more significant correlation with gene expression than promoters. Two genes that demonstrated a strong correlation of distal element accessibility changes with gene expression were the MHC class II transactivator *Ciita*^30–32^ and *Aicda*, which encodes AID^33^, the factor required for somatic hypermutation and class switch recombination. *Ciita* was down regulated upon differentiation at Div8+, yet maintained promoter accessibility, despite the acquisition of repressive histone modifications^34^ (Fig. 4c, d). Three intronic *Ciita* distal elements all lost accessibility between Div5 and 8+ in conjunction with the loss of expression. For *Aicda*, the distal elements and promoter both gained accessibility, peaking with gene expression at Div5. However, at Div8+, concurrent with a loss of gene expression, only the enhancers lost accessibility.

Although most distal elements showed a positive correlation with gene expression (Fig. 4b), the inverse was also true in that some distal elements that lost accessibility mapped to genes that gained expression. The *Irf4* gene contains examples of such distal elements that lose accessibility at a time point when expression is induced (Fig. 4c, d). ChIP-seq data from splenic naive B cells found the binding of the B cell lineage factors PU.1^35^ and PAX5^36^ to at least two of these elements, suggesting that these factors function to repress expression of *Irf4* in naive B cells at these sites. Such elements may be negative regulators of transcription and are potentially analogous to those described for T cell differentiation^37^. In addition to negative regulatory elements, a set of these negatively correlating distal elements could be epigenetically programmed to maintain accessibility but no longer influence gene expression.

### Undivided B cells contain primed accessible promoters

**Fig. 5:**
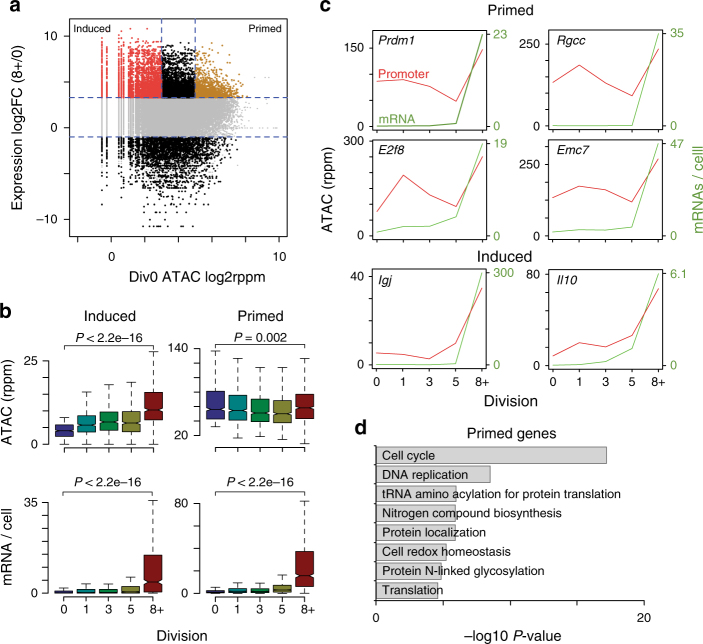
A subset of promoters display a primed accessibility structure. **a** For all genes with significant gene expression changes between Div8+ and Div0, the change in gene expression is plotted vs. their promoter accessibility at Div0. Genes in which both expression and accessibility (log2 rppm < 3) was induced are labeled in red, and genes in which expression is increased at Div8+ but were highly accessibility at Div0 (log2 rppm > 5) were labeled as primed (gold). **b** For induced and primed gene sets, the promoter accessibility and gene expression kinetics are shown across all divisions. Boxplot center line indicates data median, lower and upper bounds of boxes the 1st and 3rd quartile ranges, and whiskers the upper and lower ranges of the data. Significance between Div0 and Div8+ was determined by two-tailed Student’s *T*-test. **c** The promoter accessibility and gene expression changes across all divisions for the indicated gene promoters with a primed (top) or induced (bottom) accessibility pattern. **d** Gene ontology results for primed accessible loci in gold from **a**

Examination of accessible regions in promoters revealed that accessibility did not always correlate with gene expression at a particular division during the differentiation process. To further explore this, promoter accessibility in undivided B cells was annotated for all genes differentially expressed between Div8+ and Div0. A subset of induced genes (591) that gained expression and displayed low levels of promoter accessibility at Div0 (Fig. 5a), consistent with these genes not being expressed at Div0. These induced promoters significantly gained accessibility by Div8 (Fig. 5b), and included *Igj*, *Il10* (Fig. 5c), and *Egr1* (Supplementary Fig. 2). Unexpectedly, 820 promoters were highly accessible at Div0 despite not being expressed until Div8+ (Supplementary Data 3). These gene promoters were termed ‘primed’ because they had accessible promoters at Div0 but were not expressed at this division. Interestingly, primed genes were ultimately expressed at a higher level in Div8+ than induced genes (Fig. 5b). GO analysis of primed genes revealed enrichment for pathways that function in cell cycle processes, metabolism, translation, and protein glycosylation (Fig. 5d). Examples of genes with primed promoters include: the cell cycle regulators *E2f8* and *Rgcc*, the ER membrane component *Emc7*, and the master transcriptional regulator of plasma cell fate *Prdm1*^38, 39^.

### H3K27me3 is enriched at primed accessible promoters

**Fig. 6:**
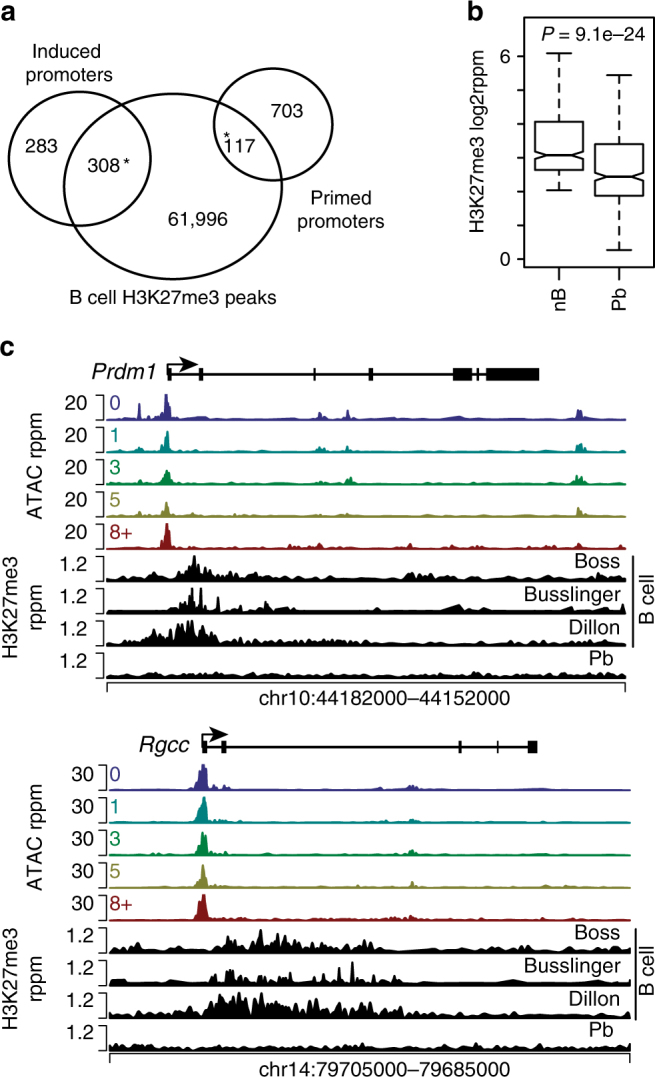
H3K27me3 marks a subset of primed-accessible promoters. **a** Venn diagram displaying the overlap of regions enriched for H3K27me3 in B cells with primed and induced accessible promoters. A significant overlap with a *P-value < 1e−5 as determined by Fisher’s exact test. **b** Box plot showing a comparison of H3K27me3 enrichment at primed-accessible promoters in B cells versus splenic plasmablasts. Boxplot center line indicates data median, lower and upper bounds of boxes the 1st and 3rd quartile ranges, and whiskers the upper and lower ranges of the data. Significance was determined by a two-tailed Student’s *T*-test. **c** Promoter accessibility across all divisions and H3K27me3 enrichment in B cells and plasmablasts are displayed for two example loci. H3K27me3 ChIP-seq data was an average of two biological replicates from both B cells and plasmablasts

The finding of primed gene promoters above, suggested that specific epigenetic mechanisms may play a role in regulating gene expression of this gene set. The trimethylation of histone H3 at lysine 27 (H3K27me3) functions to repress transcription during cell fate transitions during development and is deposited by EZH2^8, 9^. EZH2 is known to repress plasma cell genes, including *Prdm1*, in the germinal center^11–13^. To determine if EZH2 performed a similar function at primed gene promoters, ChIP-seq was performed for H3K27me3 in naive CD43^−^ splenic B cells (nB) and in CD138^+^ splenic Pb isolated at day 3 following LPS inoculation^40^. These cells represent comparable time points for Div0 (nB) and Div8+ (Pb), respectively. In total, 117 primed promoters were enriched for H3K27me3 in B cells (Fig. 6a). A strong correlation of H3K27me3 enrichment at primed promoters was also observed using two previously published datasets from B cells^36, 41^ isolated from similar splenic compartments (Supplementary Fig. 4a), confirming the programming of this gene set. Additionally, 308 induced gene promoters were marked by H3K27me3 in nB. Consistent with primed genes being expressed in Pb a significant loss in H3K27me3 was observed in Pb at primed promoters (Fig. 6b). For example, *Rgcc* and *Prdm1* were not expressed at Div0 but exhibited accessible promoters in all divisions (Fig. 6c). Additionally, surrounding the promoter accessibility peak, all three datasets showed an enrichment of H3K27me3 in B cells that was lost in Pb. In contrast, the HoxA locus, which is extensively enriched for H3K27me3, was unchanged in both B cells and Pb (Supplementary Fig. 4b), demonstrating that loss of H3K27me3 at the primed gene promoters was specific.

### Inhibition of EZH2 enhances ex vivo plasma cell formation

**Fig. 7:**
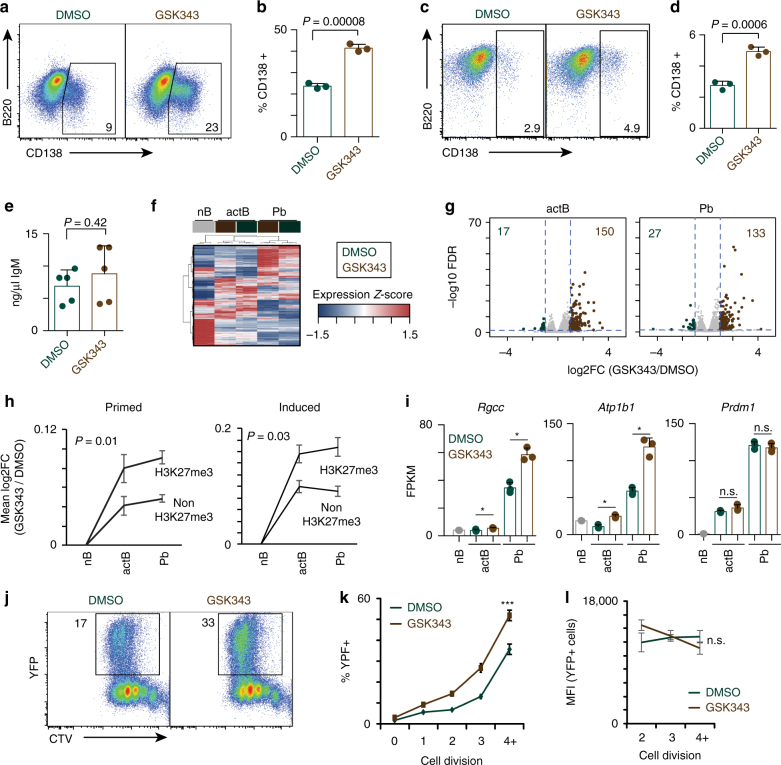
Inhibition of EZH2 results in enhanced plasma cell differentiation. **a** Representative flow cytometry analysis of B220 versus CD138 expression following ex vivo culture with LPS, IL2, and IL5 for three days +/− GSK343. **b** Quantification of data from **a**. **c** Representative flow cytometry analysis of B220 versus CD138 expression following ex vivo culture with CD40L, IL4, and IL5 for three days +/− GSK343. **d** Quantification of data from **c**. **e** Secretion rate of IgM/hr for plasma cells cultured from **a**. **f** Hierarchical clustering of samples and DEG between GSK343 treated actB and Pb. **g** Volcano plot of RNA-seq data quantitating changes in gene expression following GSK343 treatment comparing actB cells (B220^+^GL7^+^CD138^−^) (left) and Pb (CD138^+^) (right). Genes significantly up or down regulated are color-coded. **h** For primed and induced genes, the mean log2 fold change (FC) for genes marked versus not marked by H3K27me3 is plotted. Significance between datasets was determined by two-way ANOVA. **i** RNA-seq expression data for the indicated genes for each cell type. FPKM, fragments per kilobase per million. Asterisk (*) indicates significant differential expression as determined by edgeR. **j** Representative flow cytometry of cell trace violet (CTV) labeled Blimp-1-YFP B cells after three days culture with LPS, IL-2, and IL-5 in the presence of DMSO or GSK343. The percentage of YFP^+^ cells are indicated. **k** YFP^+^ cells from **j** are quantitated at each division. Significance determined by two-way ANOVA with ***P-value <0.0001. **l** The MFI of YFP+ cells from **j** is quantitated for divisions 2 through 4+. Significance was tested by two-way ANOVA. RNA-seq in **f** was performed on three biological replicates for each cell type. For **b**, **d**, **e**, **h**, **i**, **k**, and **l** the mean is plotted and s.d. is shown. Data from **a**, **c**, **e**, and **j** were representative examples from three independent biological replicates that were performed three times. For **b**, **d**, and **e** significance was determined by two-tailed Student’s *T*-test

Naive B cells express low levels of *Ezh2* mRNA, however; when stimulated ex vivo with LPS, IL2, and IL5, a transient increase in expression is observed followed by a decrease that coincided with induction of *Prdm1* expression^30^. Therefore, this ex vivo system was used to further determine the relationship between H3K27me3 and primed genes. EZH2 has been the target of drug discovery due to the presence of activating mutations in B cell lymphomas^13^. Treatment of the P3X63ag8.653 plasmacytoma cell line with the EZH2 inhibitor GSK343^13^ resulted in a dose dependent decrease in global H3K27me3 levels but not other trimethylated residues such as H3K4me3 or total histone H3 (Supplementary Fig. 4c) that is consistent with previous observations^13, 15^. Therefore, ex vivo B cell differentiation with LPS, IL2, and IL5^30^ and either DMSO or 2 μm GSK343 was used to determine the function of EZH2. Compared to control cultures, B cells cultured in the presence of GSK343 demonstrated a 2.5-fold enhancement in CD138^+^ plasma cells (Fig. 7a, b). The enhancement was not specific to LPS stimulation as cells treated with CD40L, IL4, and IL5 also displayed a similar increase in CD138^+^ cells (Fig. 7c, d). These results are consistent with enhanced numbers of CD138^+^ plasma cells from *Ezh2*^−/−^ B cells following ex vivo CD40L, IL4, and BAFF stimulation^11^. Next, following three days culture with LPS, IL2, and IL5, 1 × 10^6^ CD138^+^ cells were cultured for five hours and the total secreted IgM levels measured by ELISA. Plasma cells that formed in the presence of GSK343 did not secrete significantly more IgM than control cultures (Fig. 7e), indicating GSK343 enhanced the number of plasma cells but not their function. These data indicate that in this in vitro system, EZH2 functions as a repressor of plasma cell formation.

### Accessible promoters regulated by EZH2

To further analyze the molecular changes induced upon inhibition of EZH2, RNA-seq was performed on nB and purified activated B (actB, B220^+^GL7^+^CD138^−^) cells and Pb following 3 days culture with LPS, IL2, and IL5 in the presence or absence of GSK343. Consistent with a repressive role for EZH2, more genes were upregulated following GSK343 treatment than downregulated in both actB and Pb (Fig. 7f, g). A subset of Blimp-1 target genes require EZH2 for maximal repression, and similar to previous reports^15^, were upregulated in Pb treated with GSK343 and included *Hck and Gsn* (Supplementary Fig. 4d). The presence of H3K27me3 at primed and induced promoters predicts that the expression of these genes would be significantly altered by inhibition of EZH2. At primed genes marked by H3K27me3, a larger increase in expression was observed in both actB and Pb versus those primed genes not marked by H3K27me3 (Fig. 7h). Primed accessible genes that demonstrated enhanced expression included *Rgcc* and *Atp1b1* (Fig. 7i). Similar to the primed gene set, induced genes marked by H3K27me3 were significantly more highly induced following GSK343 treatment than induced genes not marked by H3K27me3 (Fig. 7h). These data indicate that a distinct set of primed and induced accessible promoters are regulated by EZH2 in B cells.

### Altered kinetics of *Prdm1* expression in the absence of EZH2

*Prdm1* expression is fully induced at Div8+ in vivo^2^ and at day 2 in this ex vivo culture system^30^. In purified cell types *Prdm1*, which was annotated as a primed gene, did not exhibit significant expression changes following GSK343 treatment (Fig. 7l). However, this analysis did not rule out differences in the timing of expression across cell division and differentiation. To determine a correlation between EZH2 inhibition, cell division, and gene expression, B cells from the Blimp-1-YFP reporter mouse^42^ were labeled with CTV and cultured ex vivo with LPS, IL2, and IL5 in the presence or absence of GSK343. In control cultures, YFP^+^ cells peaked after three-four divisions (Fig. 7j). In GSK343 treated cultures, YFP^+^ cells were significantly more abundant as early as division two and resulted in 2-fold more YFP^+^ cells in the culture (Fig. 7j, k). However, like the RNA expression data for *Prdm1* (Fig. 7i), the MFI for Blimp-1-YFP positive cells remained unchanged irrespective of division (Fig. 7l). These data suggest that by inhibiting EZH2, a higher proportion of cells have induced Blimp-1 expression and potentially other primed genes at earlier divisions. Thus, EZH2 may participate through multiple mechanisms to regulate the timing and magnitude of both primed and induced genes.

## Discussion

B cell differentiation is mechanistically linked to cellular division^43–45^; however, a limited toolkit exists to dissect epigenetic remodeling events in tandem with division. The sensitivity of ATAC-seq, coupled with in vivo tracking of cellular division and differentiation, identified a collection of *cis*-regulatory elements that occur during in vivo B cell differentiation to plasma cells. For example, ATAC-seq of the *Ciita* gene locus, which has been extensively studied by our lab and others^34, 46–48^, identified a larger number of potential regulatory regions than previously thought. It is important to recognize that accessibility is a readout of chromatin structure and that multiple mechanisms, including DNA methylation and the posttranslational modifications of histones, as well as the binding of transcription factors, participate in controlling accessibility and ultimately gene regulation. The integration of DNA methylation and accessibility data provided a more complete picture of epigenetic regulation of B cell differentiation. For example, in contrast to the targeted DNA hypomethylation that occurs during B cell differentiation, roughly equal numbers of loci gained or lost accessibility across the divisions, and these changes were more strongly correlated with gene expression, particularly at distal elements. Because de novo DNA methylation is very low in response to LPS, the losses in chromatin accessibility at *cis*-elements suggest additional mechanisms that facilitate gene silencing, such as repressive post-translational histone modifications. As discussed below, chromatin accessibility does not always correlate with transcriptional status. Thus, it is essential that epigenetic datasets are integrated with RNA-seq data to properly correlate chromatin accessibility, particularly at promoters, with activity of a given gene or gene system.

At the cell division/differentiation stages assayed here, the ATAC-seq and transcription data suggest several potential paths that B cells undergo as they differentiate into plasma cells. In one model, B cell differentiation and programming is progressively linked to cell division and can be divided into three molecular stages. Immediately following stimulation, few changes were observed in either chromatin accessibility or gene expression. This suggests that B cells are pre-programmed to initiate division and expand in response to type I T independent signaling events such as LPS. The second stage, from Div3-Div5, marks early B cell reprogramming events that could be a consequence of proliferation or a prerequisite for later plasma cell differentiation. During these divisions, B cells expressed the GL7 activation marker, were rapidly proliferating, and displayed initial chromatin accessibility and transcriptional changes, including global transcriptome amplification^2^. These changes are likely mediated by the LPS signaling cascade through MyD88 and TRIF, resulting in activation of NF-κB, IRF, and AP-1 transcription factors^49^. The signature of these transcription factor networks was observed in the accessibility at NF-κB and composite IRF:AP-1 motifs at Div3 and Div5 (Supplementary Fig. 1). DNA methylation was also lost in regions surrounding these motifs at the same divisions^2^. Additionally, at Div5, low levels of *Prdm1* expression can first be detected, suggesting that cells expressing Blimp-1 may be transitioning from the activated state to plasma cells. The third stage, which was assessed at Div8+ here, coincided with differentiation processes and was manifested by acquisition of CD138 and loss of B220 surface expression and full expression of *Prdm1*. This final stage resulted in the greatest changes in chromatin accessibility. A distinct switch in transcriptional networks was also observed as DNA binding motifs for essential plasma cell regulators IRF4^24, 50^ and E2A^51, 52^ became accessible. Additionally, at Div8+ a subset of sites containing activation factor motifs (i.e., NF-κB), as well as B cell lineage factors such as EBF1 lost accessibility, thus terminating the activation and B cell programs. The key plasma cell genes, Blimp-1 and IRF4 were fully induced at this stage. At this point it is unclear if the abrupt differentiation observed at division eight is programmed due to proliferation and cellular division^43–45^, changing concentrations of transcription factors^50, 53^, or through orchestrated changes in chromatin accessibility at enhancers/silencers and promoters of key B cell and plasma cell genes.

The data presented do not distinguish from an alternative model that could include a stochastic process in which the more divisions a cell undergoes the more likely it will differentiate to plasma cells. Such a model has been presented previously^44^. Another possibility is that the cells could acquire plasmablast markers (CD138) at an earlier division and continue to divide, placing them in Div8+ at the time of the assay. This model could include early expression of Blimp-1 and display an accelerated reprogramming from the B cell fate. Irrespective of the path that the cells take, the changes in accessibility at transcription factor motifs provides a roadmap to the steps required for differentiation.

The licensing of *cis*-elements, such as enhancers, is dependent on pioneering and/or cell lineage transcription factors that can displace nucleosomes at key genes^28, 54, 55^; however, it is less clear how the opposite process occurs when *cis*-element accessibility is lost and gene repression occurs. Conceptually, this could be accomplished by non-mutually exclusive processes that include loss of an activating transcription factor or the binding of a repressor that could decommission a region or an enhancer. This concept was explored using the relationship between transcription factor expression and accessibility at its putative binding sites. As somewhat expected, transcription factors that bound motifs enriched in loci that gained accessibility also increased in their expression. In contrast, transcription factor binding sites that showed decreased accessibility were also found to have increased expression of the potential binding factor. This latter point was highlighted by both the AP-1 factor ATF1 and Blimp-1. These data suggest that loss of chromatin accessibility at distinct sites may be an active process mediated by repressors. For example, in CD8 T cells, Blimp-1 binding leads to the eviction of NFATC1 and accumulation of repressive histone marks to repress PD-1 expression^56^, suggesting a mechanism by which removal of positively acting factors results in chromatin closing and gene silencing.

The kinetic analysis of gene expression and accessibility described herein revealed a subset of primed genes that contained accessible promoters but were not transcribed in undivided B cells. In memory CD8 T cells, genes with primed promoters encoded important effector functions that would be required during reactivation^37^. In naive B cells, primed promoters occurred at genes with cell cycle and metabolic functions but also the essential plasma cell transcription factor *Prdm1*^38, 39^. Therefore, similar to CD8 T cells, B cell genes that function in the next developmental phase (i.e. activation and differentiation) displayed a pre-existing chromatin accessibility programming that may reduce the number of molecular steps necessary for or allow for a more rapid induction. A subset of primed promoters was marked by the repressive post-translational histone modification H3K27me3 and inhibition of the enzyme responsible for deposition, EZH2, resulted in enhanced expression of primed genes. H3K27me3 has been shown to mark essential developmental gene promoters and has been suggested to allow precise temporal and synchronized control of transcription^8, 14, 57^. EZH2 is highly upregulated in proliferating GC B cells^11–13^ and in response to LPS in vivo^2^ and ex vivo^30^. These data suggest that in proliferating B cells, as in the GC, EZH2 functions to repress differentiation. Moreover, inhibition of EZH2 catalytic activity resulted in early induction of *Prdm1* expression ex vivo. Reducing the molecular steps necessary for induction of genes such as *Prdm1* is likely an important mechanism to allow rapid differentiation and formation of plasma cells^2^. As methods improve to analyze the histone code on small numbers of primary cells, it will be revealing to track changes in post-translational histone modifications by cell division, including H3K27me3, to precisely determine when these marks change during differentiation.

Together, these chromatin accessibility data provide a readout of the *trans*-acting factors and epigenetic mechanisms and define the chromatin centric reprogramming steps required for B cells to differentiation into plasma cells. The initiation of the plasma cell gene expression program involves an elaborate interaction of transcription factors and the opening of thousands of enhancers. Histone modifications add an important layer of complexity and provide potential temporal control mechanisms for gene expression kinetics. Defining the epigenetic landscape in a cell-division linked manner for other stimuli will provide insights into the common and unique programming of B cell fates and the control of humoral immunity.

## Methods

### Mice and cell isolation

C57BL/6 J wild-type (Stock# 000664), μMT (Stock# 002288), and CD45.1 congenic (Stock# 002014) mice were purchased from Jackson Labs. Blimp-1-YFP mice were a gift of Dr. Sue Kaech^42^. All experimental mice were between 6 and 9 weeks of age. CD43^−^ splenic B cells were purified from naive mice by magnetic purification according to the manufacturer’s instructions (Miltenyi Biotech). To induce B cell differentiation, 50 μg LPS (Enzo Life Sciences, ALX-581-008) was i.v. injected into naive mice and three days later CD138^+^ splenic plasmablasts were isolated by FACS^2^. For division labeling, purified B cells were stained with 2 μM CFSE (Tonbo Biosciences) for 8 min or 5 μM CTV (ThermoFisher Scientific) for 20 min at room temperature protected from light. CFSE staining was quenched with 1 volume FBS, and for both dyes, cells were washed with 5 volumes B cell media (RPMI 1640, 10% heat-inactivated FBS, 10 mM HEPES, 1 mM sodium pyruvate, 1× non-essential amino acids, 1× PeneStrep, 0.05 mM 2-ME), and either cultured or adoptively transferred into μMT hosts. For in vivo LPS challenge, one day post adoptive transfer mice were injected i.v. with 50 μg LPS as above. All animal experiments were approved by the Emory University Institutional Animal Care and Use Committee.

### Cell culture and ex vivo differentiation

For LPS stimulation, CD43^−^ splenic B cells were seeded at 5 × 10^5^ cells/ml in B cell media containing LPS (20 μg/ml, Sigma Aldrich), IL-2 (20 ng/ml, eBioscience), and IL-5 (5 ng/ml, eBioscience). Each subsequent 24 h the culture was supplemented with a 0.5× dose of LPS, IL-2, and IL-5 for the duration of the time course^30^. For CD40L stimulation, cells were seeded at 5 × 10^5^ cells/ml in B cell media containing CD40L (200 ng/ml, R&D Systems), IL-5 (5 ng/ml) and IL-4 (20 ng/ml, R&D Systems). Each subsequent day the culture was supplemented with a 0.5× dose of CD40L, IL-5, and IL-4 for the duration of the time course. GSK343 was a gift of Mike McCabe^13^. GSK343 was resuspended in DMSO and added to the culture at the indicated doses. P3X63Ag8.653 cells (ATCC, CRL-1580) were cultured in RPMI 1640 supplemented with 10% heat-inactivated FBS.

### Flow cytometry

Cells were resuspended at a concentration of 10^6^/100 μl in FACS buffer (1x PBS, EDTA, 1% BSA) and first stained for 15 min on ice with Fc block (BD Biosciences, #553142). Next, antibody cocktails were added for a further 15 min on ice. Following staining, cells were washed with 10 volumes 1× with FACS buffer. The following antibodies were used in this study and are from BD Biosciences: CD138^−^BV711 (#563193, Clone 281-2, 1:200); and Tonbo Biosciences: B220-PE-Cy7 (#60-0452, Clone RA3-6B2, 1:400), CD11b-FITC (#35-0112, Clone M1/70, 1:100), Viability Ghost Dye-APC-Cy7 (#13-0865-T100, 1:100). Analysis was performed on a LSRII and sorting was performed on an ARIAII instrument (BD Biosciences).

### Ab secretion assay

Day 3 LPS cultures were harvested, washed, and the percentage of CD138^+^ cells determined by flow cytometry. For each sample, 1 × 10^6^ CD138^+^ cells were seeded in 1 ml fresh B cell media for 5 h. Secreted Ab was determined by anti-IgM ELISA (Southern Biotech).

### ATAC-seq

ATAC-seq^17^ was performed using 20,000 FACS isolated cells that were resuspended in nuclei lysis buffer (10 mM Tris pH 7.4, 10 mM NaCl, 3 mM MgCl_2_, 0.1% IGEPAL CA-630) and centrifuged at 500×*g* for 30 min. Next, nuclei were transposed in 25 μl tagmentation reaction mix (2 × Tagmentation buffer and 2.5 μl Tagmentation Enzyme from the Illumina Nextera DNA Library Prep Kit) at 37 °C for 1 hr, diluted 2 × in Lysis Buffer (300 mM NaCl, 100 mM EDTA, 0.6% SDS, 1.6 μg Proteinase-K), and incubated for 30 min at 40 °C. Low-molecular weight transposed DNA was isolated by size selection using SPRI-beads and PCR amplified using 2 × KAPA HiFi HotStart Ready mix and Nextera Indexing Primers (Illumina, Inc). A second size-selection was performed post-PCR to enrich for low molecular weight DNA. Samples were quality checked for ATAC-seq specific patterning on a bioanalyzer and were pooled at an equimolar ratio and sequenced on a HiSeq2500 using 50 bp single-end chemistry.

### ChIP-seq

ChIP assays^58^ were performed using 10 × 10^6^ CD43^−^ splenic B cells or 1 × 10^6^ CD138^+^ splenic plasmablasts for each immunoprecipitation. Samples were fixed in 1% formaldehyde for 10 min, chromatin isolated, and sonicated to an average size of 400 bp. One microgram of anti-H3K4me3 (ab8580, Abcam) or anti-H3K27ac (39133, Active Motif) was prebound to Dynal Protein G magnetic beads (ThermoFisher Scientific) and DNA-chromatin complexes immunoprecipitated overnight at 4 °C. DNA was reverse cross-linked and purified using a PCR cleanup kit (Qiagen). ChIP-DNA was diluted 1:20 and enrichment of the *Hoxa9* (Hoxa9-F: 5′-GTGGTGGTGGTGATGATACA; Hoxa9-R: 5′-TTGCAGCTTCCAGTCCAA) and *Actb* (Actb-F: 5′-AGCCAACTTTACGCCTAGCGT; Actb-R: 5′-TCTCAAGATGGACCTAATACGGC) loci tested by qPCR. Remaining ChIP-DNA was used as input for the KAPA HyperPrep kit (KAPA Biosystems, Inc). ChIP-seq libraries were sequenced on a HiSeq2500 using 50 bp paired-end chemistry.

### ATAC-seq and ChIP-seq data processing

Raw sequencing reads were mapped to the mm9 version of the mouse genome using Bowtie^59^. Uniquely mappable and non-redundant reads were used for subsequent analyses. HOMER^35^ software was used for peak calling, gene annotation, and to annotate transcription factor motifs in DAR. Data were normalized to reads per peak per million^17^ using Eq. (1).

$${\mathrm{rppm}} = {\mathrm{Reads}} \times \left( {\frac{{1 \times 10^6}}{{{\mathrm{Unique}}\,{\mathrm{reads}} \times {\mathrm{FRiP}}}}} \right),$$

H3K27me3 ChIP-seq datasets from Boss GSE97695^40^, Busslinger^36^ GSE38046 and Dillon^41^ GSE42706 labs were downloaded and mapped, annotated, and normalized as detailed above. To map enhancers to genes, DAR were annotated to the nearest transcription start site and had to occur within a window of greater than 2 kb and less than or equal to 100 kb. Division specific DML were previously reported^2^. The overlap of DML and ATAC-seq DAR were determined using bedtools v2.24.0^60^. Tissue specific enhancer datasets were computed previously^2^ and the odds ratio and significance of overlap determined using a Fisher’s exact test.

### RNA-seq

Total RNA was isolated from splenic naive CD43^−^ B cells and FACS isolated B220^+^GL7^+^CD138^−^ activated B cells and CD138^+^ Pb from ex vivo B cell cultures treated with GSK343 or DMSO using the Qiagen RNeasy kit. For library generation, 500 ng of RNA was used as input for the KAPA Stranded mRNA-seq kit with mRNA capture beads (KAPA Biosystems, Inc). Libraries were quality checked on an Agilent Bioanalyzer, pooled at an equimolar ratio and sequenced on a HiSeq2500 using 50 bp paired-end chemistry. Raw sequencing reads were mapped to the mm9 genome using TopHat2^61^ and the UCSC mm9 Known Gene reference transcript database^62^. For each sample, reads that overlapped exons of unique ENTREZ genes were annotated using the GenomicRanges (v1.22.4) package in R/Bioconductor. Genes with less than 3 reads per million in at least 3 samples were removed and edgeR^63^ used to find significantly differentially expressed genes between GSK343 and DMSO conditions. Genes with an FDR < 0.05 were termed significant.

### Immunoblotting

P3X63Ag8 cells were lysed on ice in RIPA buffer (150 mM NaCl, 5 mM EDTA pH 8.0, 0.5% sodium deoxycholate, 0.1% SDS, 1% IGEPAL, 20% glycerol, 50 mM Tris pH 8.0) for 30 min. Protein concentration was determined by Bradford assay and 75 µg whole cell extract was loaded for each sample. Immunoblotting was performed under standard conditions using the following antibodies sequentially probed on the same blot: histone H3 (600-401-879, Rockland Immunochemicals), H3K4me3 (ab8580, Abcam), and H3K27me3 (07-449, EMD Millipore). Raw images of the blots can be found in Supplementary Fig. 5.

### Data availability

All sequencing data are available under accession GSE97698 at the NCBI Gene Expression Omnibus. All data analysis code is available from the corresponding authors upon request.

## Electronic supplementary material

Supplementary Information

Description of Additional Supplementary Files

Supplementary Data 1

Supplementary Data 2

Supplementary Data 3
