# Supplementary material for: Plasma cell differentiation is controlled by multiple cell division-coupled epigenetic programs
Source: Nat Commun. 2018 Apr 27;9:1698. doi: 10.1038/s41467-018-04125-8 (PMC5923265; doi:10.1038/s41467-018-04125-8)
Supplement: Supplementary file 1 — Supplementary Information [file 41467_2018_4125_MOESM1_ESM.pdf]

Plasma cell differentiation is controlled by multiple cell division-coupled epigenetic programs

Scharer et al.

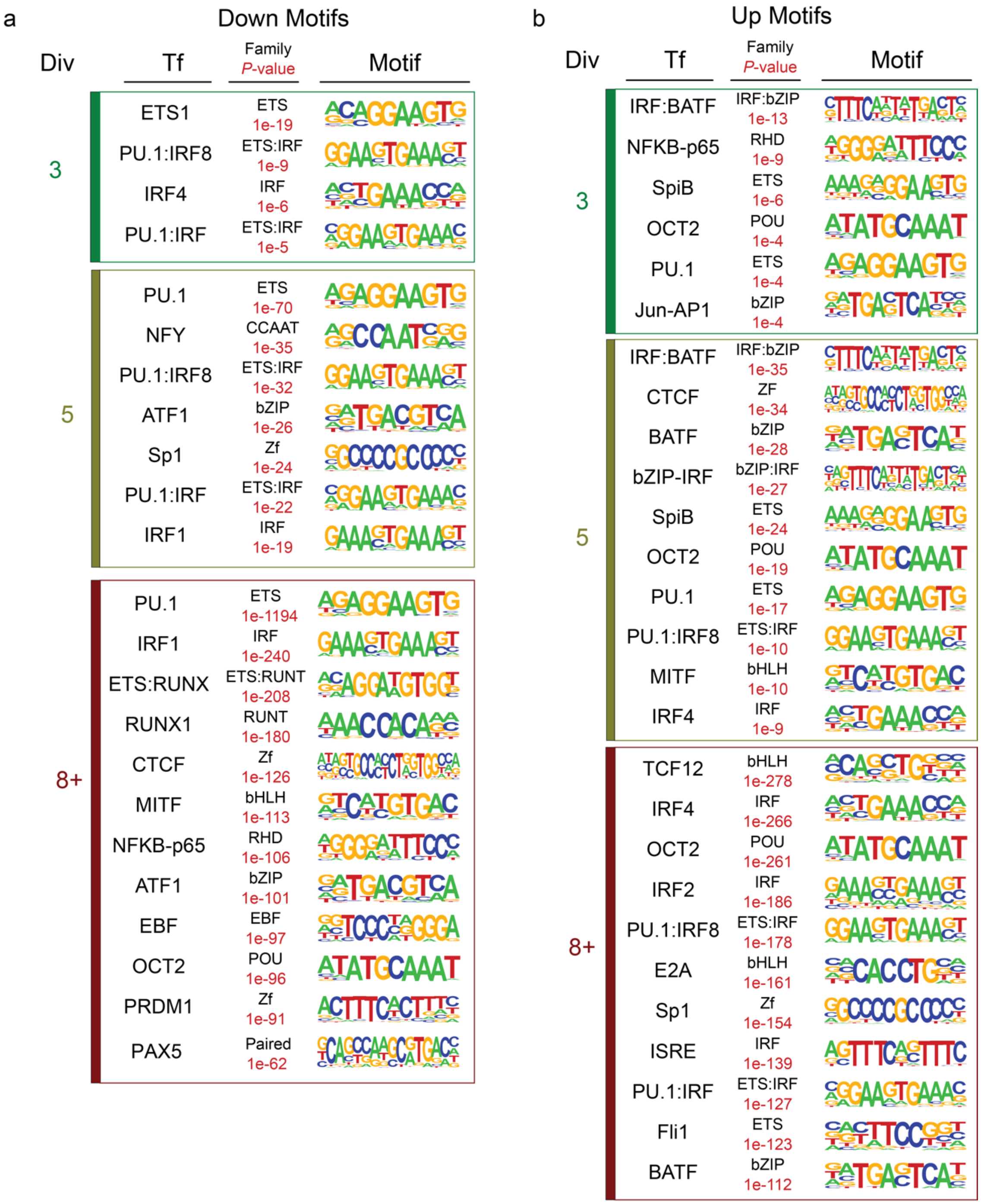

Supplementary Figure 1. Transcription factor motifs enriched in DAR. Enriched transcription factor motifs for significant increases **(a)** and decreases **(b)** in accessibility identified in Figure 1d. The transcription factor family, enrichment *P*-value, and consensus binding sequence identified are indicated.

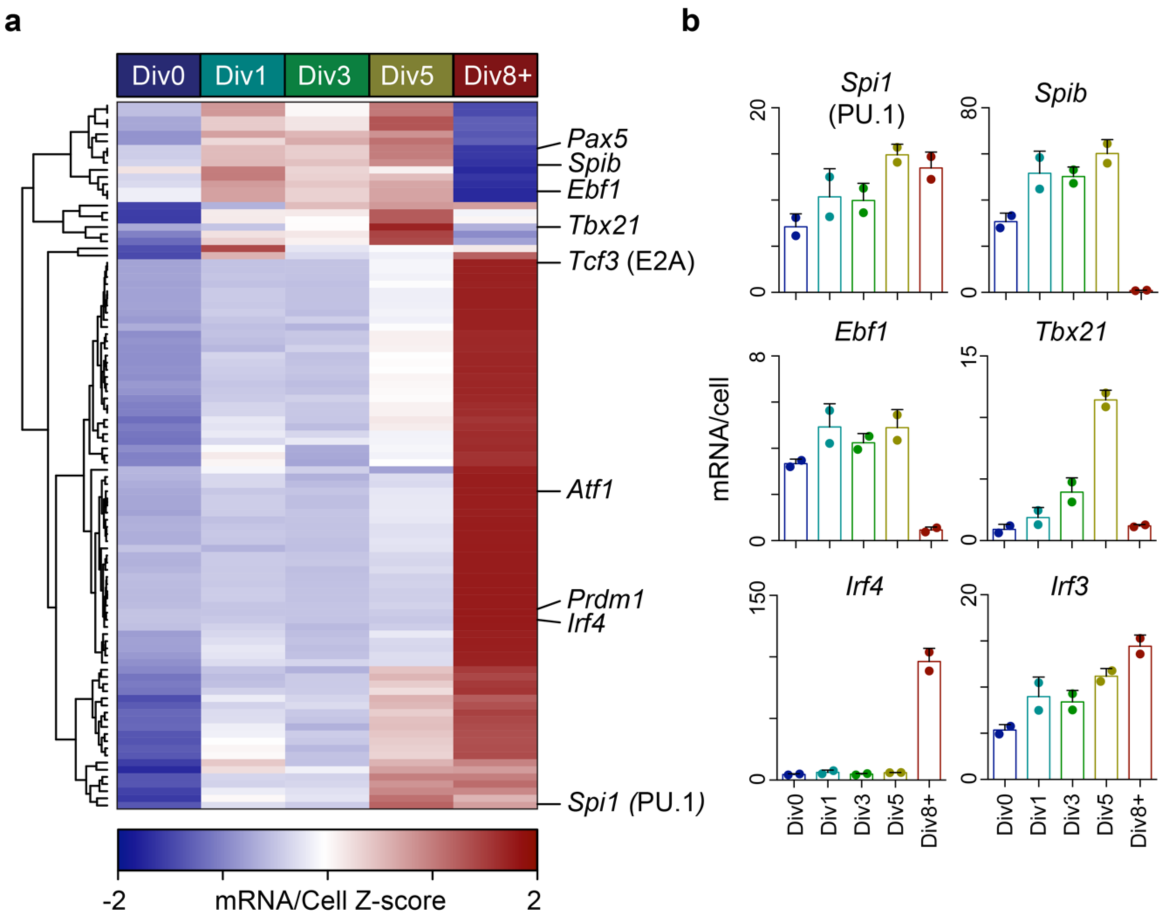

Supplementary Figure 2. Gene expression dynamics of transcription factors predicted to bind DAR. (a) A Heatmap showing the change in gene expression at each division for 99 transcription factors expressed at > 1 mRNA/cell. **(b)** Gene expression of select genes from (a) is shown at each division. Data are summarized as mean of two biological replicates and standard deviation is shown.

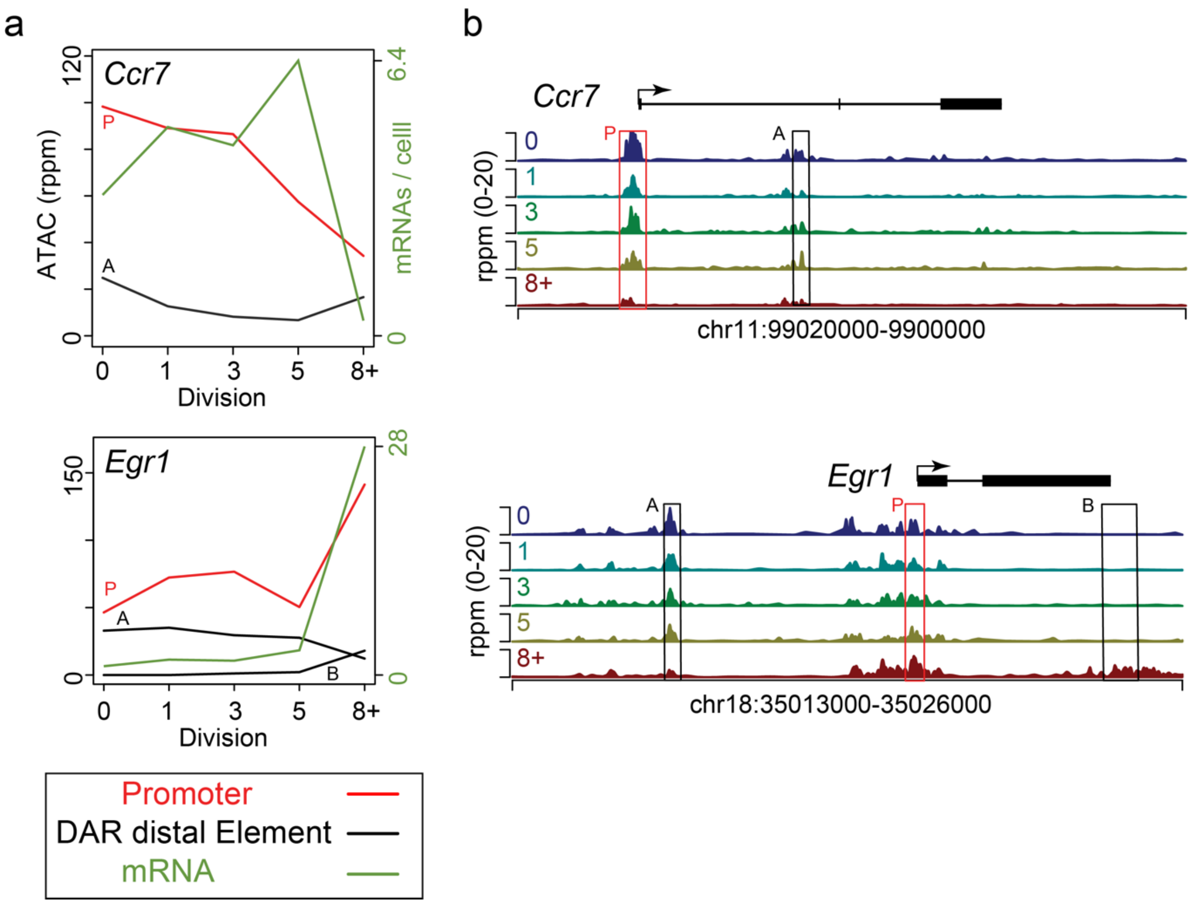

Supplementary Figure 3. Correlation changes in gene expression and promoter accessibility. (a) The changes in accessibility at distal elements (black lines) and promoters (red line) are compared to the changes in gene expression (green line) for the indicated gene. **(b)** ATAC-seq accessibility profile for each gene plotted in (a).

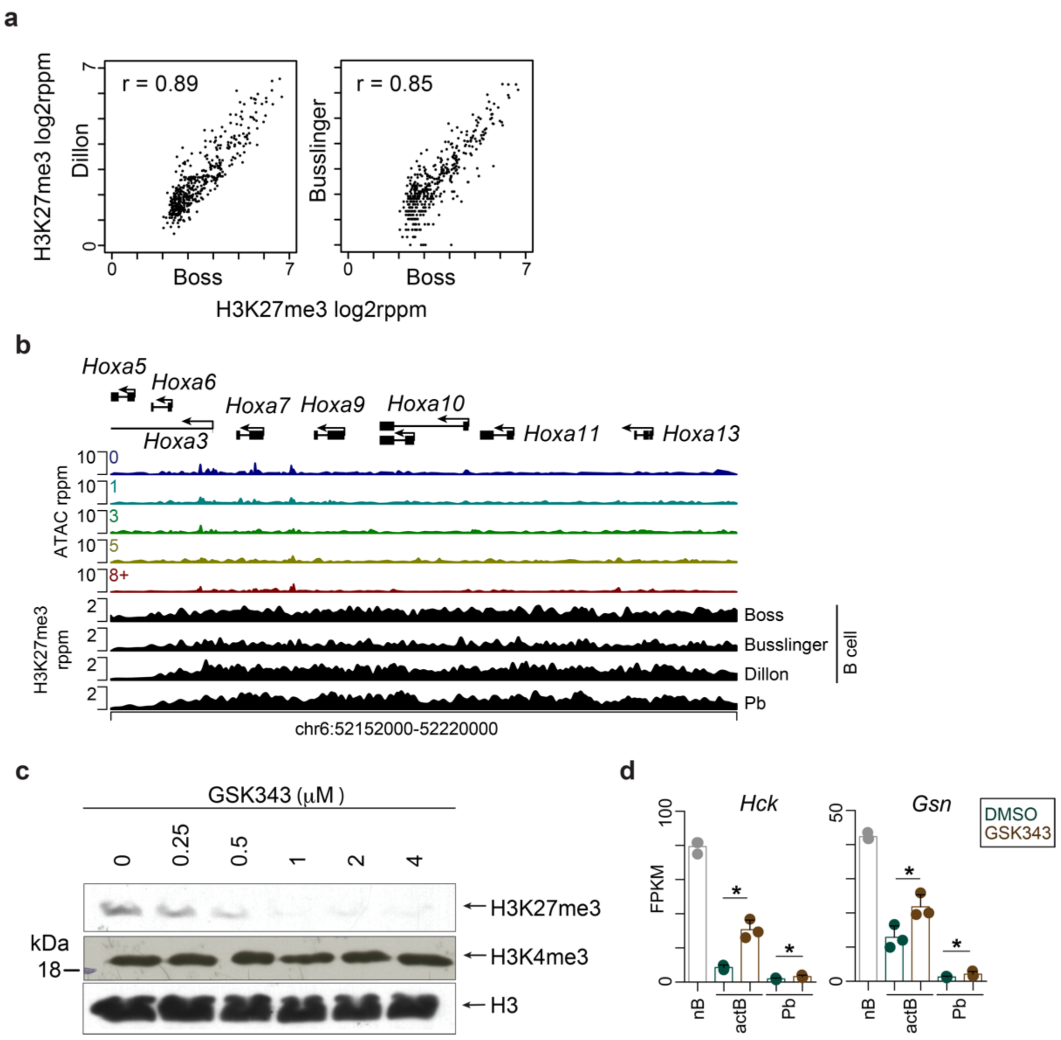

Supplementary Figure 4. GSK343 enhances the expression of primed accessible genes. **(a)** The correlation of H3K27me3 signal in B cells from data generated by the Boss lab[40] versus Dillon[41] (left) or Busslinger[36] (right) datasets. Pearson's correlation r-value for each comparison is displayed. **(b)** Genome plot of the HoxA locus showing the ATAC-seq accessibility at each division and the H3K27me3 enrichment in B cell and plasmablasts. rppm, reads per peak per million. **(c)** Western blot analysis of P3X63Ag8.653 cells treated with the indicated dose of GSK343 after 3 days culture. **(d)** RNA-seq expression data for the indicated genes for each cell type. FPKM, fragments per kilobase per million. * indicates significant differential expression as determine by edgeR. The mean and standard deviation of three biological replicates is plotted.

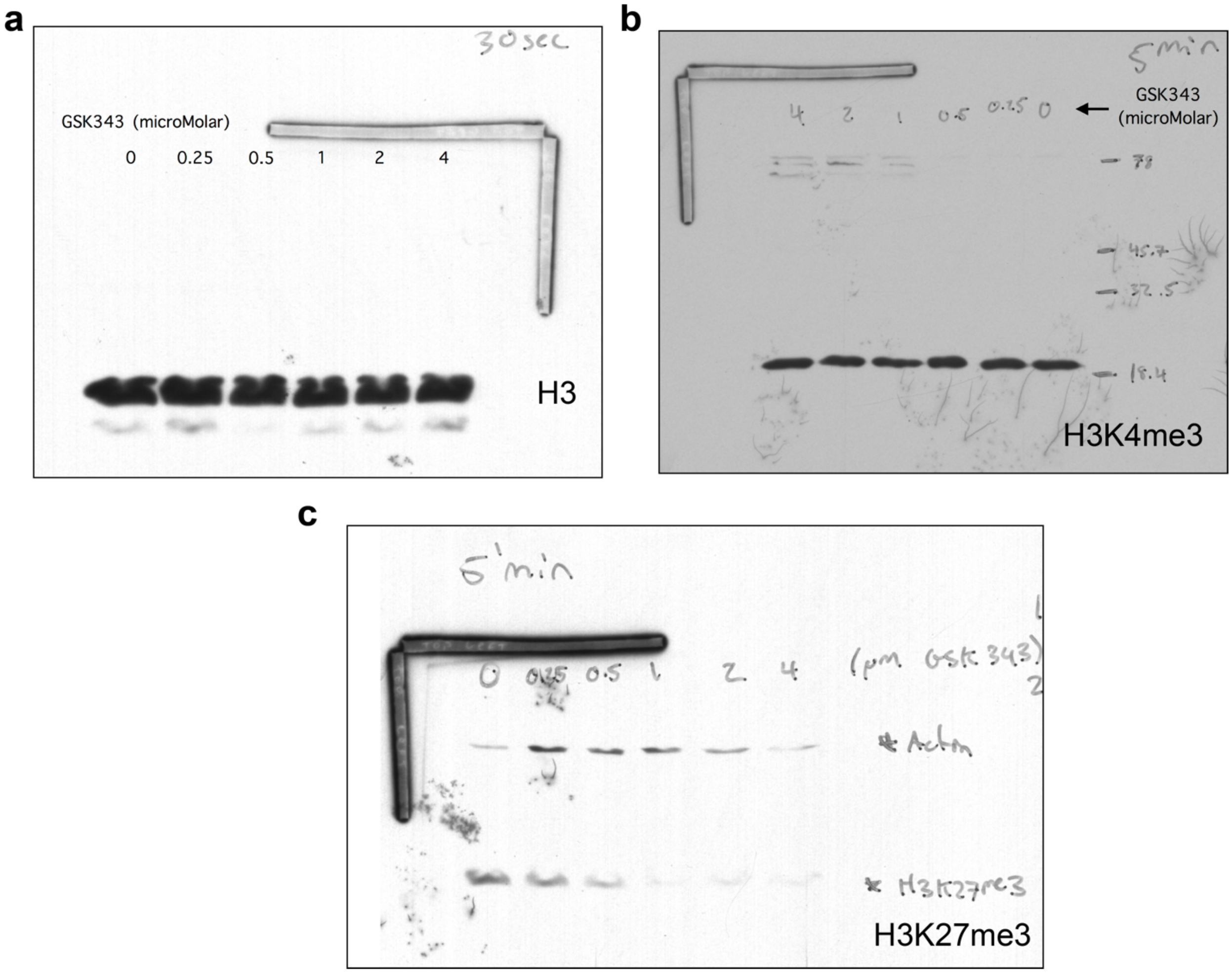

Supplementary Figure 5. Raw western blot images. Western blot images to accompany Supplementary Fig. 4c. The blot was probed with antibodies against (a) anti-histone H3, (b) anti-histone H3 lysine 4 trimethylation (H3K4me3), and (c) anti-histone H3 lysine 27 trimethylation (H3K27me3). Each blot is annotated with the sample loaded in each lane, exposure time, and location of correct band.
