# Supplementary material for: Plasma cell differentiation is controlled by multiple cell division-coupled epigenetic programs
Source: Nat Commun. 2018 Apr 27;9:1698. doi: 10.1038/s41467-018-04125-8 (PMC5923265; doi:10.1038/s41467-018-04125-8)
Supplement: Supplementary file 2 — Description of Additional Supplementary Files [file 41467_2018_4125_MOESM2_ESM.pdf]

Description of Additional Supplementary Files

File Name: Supplementary Data 1
Description: Division linked differentially accessible loci (DAR).

File Name: Supplementary Data 2
Description: Expressed transcription factors with enriched motifs in DAR.

File Name: Supplementary Data 3
Description: Primed accessible promoters.
